# Cytomegalovirus in Pregnancy: Effects on the Developing Embryo and Fetus, Diagnosis and Treatment: Where to Go Now? A Narrative Review

**DOI:** 10.3390/ijms27010252

**Published:** 2025-12-25

**Authors:** Asher Ornoy, Liza Weinstein-Fudim

**Affiliations:** 1 Adelson School of Medicine, Ariel University, Ariel 40700, Israel; 2 Program for the Development of Children with Special Needs, Jerusalem Multidisciplinary College, Jerusalem 9422408, Israel; 3 Department of Medical Neurobiology, Hebrew University Hadassah Medical School, Jerusalem 91120, Israel

**Keywords:** cytomegalovirus, congenital infection, valacyclovir, immunoglobulin, vaccine, neonatal screening

## Abstract

Cytomegalovirus (CMV) is the most common infectious cause of congenital malformations, often presenting with atypical clinical findings. Fetal damage is most severe following primary maternal infection during the first trimester of pregnancy, with the likelihood of transmission increasing with pregnancy advancement. CMV damage may continue to intensify during the early postnatal years. In this narrative review we summarized publications from the last 30 years addressing the epidemiology, diagnosis, prevention and treatment of CMV in pregnancy, with a special emphasis on embryonic and fetal damage. Substantial progress has been made in the diagnosis and treatment of CMV infection during pregnancy, warranting a reconsideration of current clinical approaches. Assessment of viral load enables prediction of fetal infection; its reduction by maternal treatment with valacyclovir may lower both the rate and severity of transmission. Confirmed fetal infection can be diagnosed by amniocentesis and viral DNA detection. Clinical manifestations in infants may be evident at birth (cCMV) or gradually emerge during the first years. The most common fetal damage is hearing loss alongside a variety of brain lesions resulting in significant neurological deficits, including intellectual impairment. Brain involvement is diagnosed by ultrasound or magnetic resonance imaging (MRI). Pharmacological treatment with ganciclovir or valganciclovir, if initiated early after birth, can slow the progression of hearing loss and may ameliorate other neurological and neurodevelopmental deficits. As of today, there is no approved CMV vaccine for prevention. The mRNA-1647’s vaccine, currently in phase 3 clinical trial, appears promising. These advances underscore the need for screening pregnant women in the first trimester and newborn infants of mothers suspected of having CMV infection. Neurodevelopmental follow up for several years, including hearing and visual assessment, is advised in all infants positive for CMV. Infants with clinical manifestations should be offered treatment as early as possible following diagnosis of cCMV.

## 1. Introduction

Cytomegalovirus (CMV) is common and can infect individuals across all ethnic and socioeconomic groups worldwide. Among adults aged 40–50 years, seroprevalence ranges from 50% to 85%. In certain populations in Asia and Africa, especially in densely populated areas and areas of low socio-economic status, it can be as high as 100% [1]. It is the leading cause of intrauterine infection, affecting the developing embryo and fetus, and is likely the most frequent cause of sensorineural hearing loss, intellectual disability and neurological dysfunction in infants [2,3,4]. In immunocompetent individuals, CMV infection is usually asymptomatic or associated with only mild, self-limited symptoms, with no long-term sequelae. Some experience mononucleosis-like syndrome with prolonged fever and mild hepatitis. Following primary infection, CMV remains latent in the host, primarily within the salivary glands, for life. Reactivation of latent infection may occur, and reinfection with a different strain is also possible, even in immunocompetent individuals [5,6].

Humans are the unique reservoir for human CMV; however, CMV-like viruses have also been identified in multiple non-human primates and in other mammals, with evidence of host-specific strains [7,8]. There are several strains of CMV that infect humans and therefore recurrent infections by different strains are common even in immuno-competent individuals. CMV, like other herpes viruses, can remain latent after primary infection resides mainly in the salivary glands and may reactivate later in life [6,9].

CMV infection requires close contact with individuals shedding the virus, and may take place by contamination from infected body fluids, sexually, through organ transplantation, or, rarely, via blood transfusions [1,6,10,11]. Indirect transmission via contaminated objects (fomites) is also possible, posing a particular risk to pregnant women in close contact with infected children [12,13,14]. Human Herpes viruses including CMV have been found to persist in fomites up to one week [12]

In infected individuals, IgM antibodies may persist for 6–18 months after both primary and non-primary infection, complicating the diagnosis of recent infection [7,8].

Following primary infection in the first trimester of pregnancy, a lack of IgM antibodies in the second trimester or high IgG avidity does not exclude congenital CMV (cCMV) [15]. Both primary or non-primary infection (reinfection by a different strain or reactivation of a persisting strain) can lead to symptomatic cCMV, which is more common in primary infection [16,17]. First-trimester infections cause more severe sequelae including early abortions or stillbirths. However, even later in pregnancy, damage, especially hearing impairment, is possible [8,18]. As there is still no efficient immunization, cCMV is a major public health problem for pregnant women and their offspring [6].

In the last decade there have been major changes in our understanding of CMV infection in pregnancy, mainly due to possibilities to reduce, in infected women, the rate of trans-placental transmission of the virus and the development of treatment modalities. This brought in new medical recommendations for prevention, diagnosis and treatment of CMV infection in pregnancy.

The main purpose of this narrative review is to summarize current knowledge and highlight the importance of screening pregnant women and newborns for possible CMV infection. In addition, we advocate for neurodevelopmental follow-up, including hearing and vision assessments, for all infants born to mothers with first trimester primary or non-primary CMV infection. We therefore searched for original studies, position papers, metanalyses and reviews that are relevant to our aim, most of them published in the last 20 years.

## 2. Congenital CMV (cCMV)

The prevalence of fetal CMV infection (cCMV) ranges from 0.2% to 2.5% of live births, [6] with an average of approximately 0.67% [19]. Younger maternal age, single marital status, and non-White race are associated with higher cCMV rates [20,21]. Clinical findings in CMV-affected newborns may be evident at birth or may gradually emerge during the first years of life [6].

**Figure 1:**
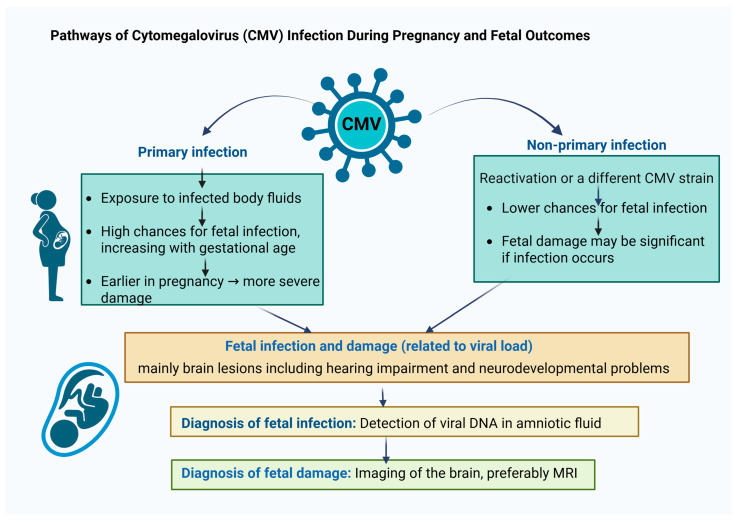
Possible outcome of primary or non-primary maternal CMV infection, possible fetal infection, antenatal diagnosis of fetal infection and possible subsequent damage.

Vertical transmission is more frequent in primary infection than in non-primary infection. However, because non-primary infections are more common, the absolute number of infants with cCMV resulting from non-primary infection is equal to or greater than that from primary infection [22,23]. Vertical transmission is thought to occur primarily via hematogenous spread: placental infection occurs first, followed by viral replication and transfer to the fetus, where the virus replicates in the renal tubular epithelium before entering the amniotic fluid [24]. Ascending CMV infection from the maternal genital tract is rare but possible. The virus, after entering the amniotic fluid, replicates in the fetal oropharynx [25]. Primary infection during the first trimester carries a significantly higher risk of symptomatic fetal involvement and severe damage, although the likelihood of transmission increases with gestational age [7,26,27]. Vertical transmission can occur even if maternal primary infection took place several months before conception [26,27,28,29], (Figure 1).

## 3. Hearing Loss Is the Most Common Neonatal Presentation of cCMV

The embryo and fetus are most susceptible to teratogens during active organogenesis, which in humans occurs in the first trimester of pregnancy. CMV can affect fetal organs even after major organogenesis, as infection and inflammation may persist for years after birth, leading to progressive damage [6,26,27,29,30]. Only 10–15% of newborns born to mothers with primary CMV infection exhibit the typical clinical manifestations of cCMV at birth [7,9,31]. They may include intrauterine growth restriction, microcephaly, hepatosplenomegaly, petechiae, jaundice, chorioretinitis, thrombocytopenia, anemia and/or other atypical findings [6,32,33,34,35,36,37]. Maternal viral load is an important predictor of neonatal cCMV: higher viral loads are associated with both increased risk and severity of neonatal symptoms [30]. In a recent meta-analysis, Salome et al. [30] demonstrated that the maternal blood and urine viral load are good predictors of neonatal cCMV and severity. However, blood is a better predictor in comparison to urine. Gilad et al. [38] in their meta-analysis on amniotic fluid viral load (only studies where viral load was defined as viral copies per ml of amniotic fluid) on 443 pregnancies with CMV infection from eight studies found that affected pregnancies had more than double amniotic fluid viral load compared to non-affected pregnancies. Moreover, the viral load was highest in fetuses with positive ultrasonographic brain imaging findings or other postnatal findings of cCMV.

The most common sequela of cCMV is sensorineural hearing loss that may be progressive due to ongoing cochlear damage [39]. Progressive hearing loss is manifested by extensive infection of the inner ear, especially affecting the epithelial cells in the organ of Corti [40,41,42,43]. It is often accompanied by various degrees of brain infection [41,42]. CMV infection of the vestibular labyrinth was also described with damage to sensory cells in the utricle and in the crista ampullaris [42,43]. As the virus resides in the inner ear for relatively long periods of time, hearing loss often tends to worsen with time. Brain involvement was observed in many children with hearing loss, mostly those with high viral loads, demonstrating a strong correlation between brain infection and cochlear damage, especially in children with early onset hearing loss [44]., Hence, brain abnormalities predict early hearing loss with 84% specificity and 43% sensitivity. Similar findings were described by Hranilovich et al. [45]. It is therefore recommended that infants with cCMV and early hearing loss undergo brain imaging studies [28]. Negative findings have almost 100% accurate predictive value. Although urine and saliva viral load in infants with cCMV infection is higher in symptomatic children in comparison to non-symptomatic children, viral load cannot predict hearing loss or vestibular damage [46,47].

Despite some controversies among the studies, it seems that the rate of hearing impairment among infants with pathological brain imaging is significantly higher compared to infants without brain imaging pathology. It is also clear that often, the hearing impairment only develops later during childhood.

It is recommended that infants born to CMV-infected mothers undergo periodic hearing assessments throughout infancy and early childhood, even when hearing is normal at birth and there are no signs of brain involvement. Similarly, periodic assessment of vision is also advised, preferably up to 5–6 years of age.

A summary of the main studies related to hearing impairment is given in Table 1.

## 4. Fetal Brain Damage and Neurological Sequelae Caused by CMV Are the Most Debilitating Complications of cCMV

Numerous brain imaging abnormalities have been described in children with cCMV. These abnormalities are best visualized by MRI but may also be detected by ultrasound. Ultrasonographic and MRI brain findings in neonates with cCMV can be used to predict the future neurodevelopmental outcomes [51].

Malinger et al. [52] summarized the typical ultrasonographic brain findings. The changes are better visualized in the second half of pregnancy, early in the second trimester it may be too soon to demonstrate most ultrasonographic findings. Of the more common findings, the authors list ventriculomegaly, microcephaly and increased periventricular echogenicity. Brain calcifications are a relatively common finding in several intrauterine infections (i.e., Toxoplasmosis, Rubella, Varicella) and are present in 21–43% of CMV cases. These are disseminated in different parts of the brain, including the basal ganglia and cerebellum, or in a periventricular location. Periventricular pseudocysts and intraventricular synechia may also be observed. Cerebellar changes are also common, generally with damage to cerebellar structures (Table 1).

Similar changes were also described by Boppana et al. [53] examining cranial CT scans. Most of the infants with abnormal CT scans at birth developed at least one sequela of cCMV, with 59% suffering from intellectual disability. The authors concluded that a cranial CT scanning is a strong predictor of adverse neurodevelopmental outcomes, because in children with normal CT scans, only 29% had neurological sequelae [53]. Other investigators [51,54] came to similar conclusions.

Fetal brain imaging by MRI seems to be superior to both ultrasound and CT and is generally used for better visualization of the human fetal brain. Mascio et al. [55] performed MRI examinations on 95 fetuses diagnosed with intrauterine CMV infection by PCR. They all exhibited normal ultrasonographic brain imaging and underwent, within 3 weeks, brain MRI. They found that 10.5% of these fetuses had various structural brain malformations not observed by ultrasound.

In conclusion, brain imaging–preferably MRI–is a valuable tool for the prediction of neurological sequelae and a high susceptibility to hearing impairment in children with symptomatic cCMV. The more common changes are ventriculomegaly, increased periventricular echogenicity, microcephaly and calcifications commonly in basal ganglia and cerebellum. We should, however, remember that neurodevelopmental sequalae and hearing impairment have also been observed in infants without any pathological brain changes at birth, and normal neurodevelopment was also observed in children with abnormal in utero brain imaging findings. Caution is required when counseling parents; it is important to address the limitations of the findings and to recommend continuous neurodevelopmental follow-up throughout childhood. Equally important is emphasizing the need for early intervention when neurodevelopmental delays are diagnosed.

## 5. What Are the More Common Neurodevelopmental Difficulties

One of the major complications of cCMV is the neuro-developmental damage, including varying degrees of intellectual disability. Learning disabilities may also occur in CMV-infected children with normal intellectual function and no other clinical symptoms of cCMV [25,55,56]. A possible association between cCMV and autism spectrum disorder (ASD) was also suggested [57,58]. However, it is impossible to accurately predict the extent of neurodevelopmental impairment at birth, as several sequelae—particularly hearing impairment and intellectual disability—may be progressive due to ongoing inflammation. The rate of neurodevelopmental problems differs among children with cCMV and those without neonatal symptoms. Keymeulen et al. [49] studied the developmental outcome of 753 children with cCMV at different ages. Of them, only 70.4% had normal development. Within their cohort, 16.9% had mild, 7.4% had moderate, and 5.2% had severe neurodevelopmental problems. Many had hearing loss. Speech and language impairment were observed even in the absence of hearing impairment. In total, 2.5% of the children had autism spectrum disorder (ASD) in comparison to 0.7% in the general population.

Barlett et al. [48] reported that 7–11% of children born with asymptomatic congenital CMV had some degree of hearing impairment, compared to 34–41% of those with symptomatic CMV at birth. The asymptomatic children had normal development. Normal development at 12 months of age, in the absence of microcephaly, makes subsequent neurodevelopmental or intellectual impairment unlikely [59]. A systematic review of children with congenital CMV who were asymptomatic at birth concluded that they have only low rates of neurodevelopmental disorders [50].

In contrast, infants with symptomatic CMV infection at birth are at high risk for severe CNS sequelae, including intellectual and motor impairment, microcephaly, sensorineural hearing loss, chorioretinitis and sometimes seizures. These sequelae evolve in the early years of life, with 45–90% experiencing these neurologic abnormalities [2,3,29,36,60].

A summary of the neurodevelopmental findings is given in Table 2.

## 6. How Can Maternal CMV Infection Be Diagnosed?

Primary infection is defined as CMV infection in a previously seronegative individual, whereas non-primary infection is defined as a significant rise in IgG or IgM antibody titers in someone with prior immunity [6].

In contrast to many primary viral or bacterial infections where IgM is elevated only for several weeks, following CMV infection, IgM antibodies may remain elevated for a relatively long period of time (6–18 months) in both primary and non-primary infections [13,14,15,22]. Hence, IgM antibody levels alone are not a reliable indicator of recent infection. Studying viral load in blood or body secretions in every pregnant woman is also impractical. In addition, CMV infection is difficult to diagnose based solely on clinical findings, which are often nonspecific or absent [6,61,62]. When both IgG and IgM antibodies are present and the previous serological status is unknown (as is often the case), distinguishing primary from non-primary infection is challenging. IgG avidity testing is a useful tool for differentiating past infection (high avidity) from recent infection (low avidity); an avidity index below 30–35% is generally considered indicative of recent primary infection. Non-primary CMV infection (reinfection or reactivation) is usually diagnosed if there is either a significant rise in IgG antibodies in a person with previous positive IgG, or positive IgG and positive IgM that were previously negative [6,15,61,62,63,64,65]. Despite these diagnostic tools, a small subset of pregnant women with both IgG and IgM antibodies cannot be definitively classified by these methods as having primary or non-primary infection.

A low avidity index, particularly when assessed early in pregnancy, reliably indicates recent infection and helps stratify fetal risk, especially if there are also fetal ultrasonographic abnormalities [15,61].

When avidity results are intermediate, or late in pregnancy, monitoring viral load (e.g., via CMV DNA detection in amniotic fluid or maternal blood) can improve diagnostic accuracy [62]. A review by Lazzarotto et al. [66] further supports the use of CMV IgG avidity testing in IgM-positive pregnant women to distinguish primary from non-primary infections. They also recommended CMV DNA testing as a secondary tool only when avidity is inconclusive or unreliable. Muller et al. [67] studied 553 serum samples from pregnant women for CMV IgG and IgM antibodies and IgG avidity and, in addition, also studied anti-CMV IgM antibodies against recombinant p52 (defines early phase of infection) and anti CMV IgG antibodies against glycoprotein B (that defines late phases of infection by ELISA. They found that these tests have a 92.8% positive prediction value, much higher than IgG avidity. They recommended these additional tests in cases where avidity testing is inconclusive.

Viral load can be used for identification of CMV infection and prediction of its severity. It can therefore be used as an additional tool to identify the severity of primary infection, but not as the sole diagnostic tool, as it cannot define primary from non-primary infection. However, it is a good diagnostic tool to identify cCMV if performed in the first 2–3 weeks after birth [68]. Studies on the importance of viral DNA detection to predict fetal effects describe viral load in amniotic fluid, maternal blood or urine. The best predictor seems to be amniotic fluid, followed by maternal blood and urine. Sapuan et al. [64] reported that CMV shedding, most commonly in cervicovaginal secretions, occurs in about 21.5% of seropositive pregnant women. Although shedding alone is not diagnostic of primary infection, it may reflect non-primary viral activity offering a useful complement to serological testing in complex cases. Together, these findings reinforce the use of IgG avidity as a first-line diagnostic tool, with CMV DNA detection serving as a valuable adjunct in the small subset of pregnancies where avidity results are non-informative. CMV IgM p52 antibodies and IgG gB antibody detection may be an additional tool.

## 7. Can Fetal CMV Infection Be Diagnosed?

The most effective method for diagnosing intrauterine fetal CMV infection is molecular detection of viral DNA [6,31,69]. Studies using RT-PCR have demonstrated a correlation between viral load in the amniotic fluid and the degree of fetal damage. Because it takes approximately 5–7 weeks from fetal infection for viral replication in the kidneys to result in sufficient viral shedding into the amniotic fluid, PCR testing is not considered reliable before the 21st week of gestation. Therefore, PCR should generally be performed no earlier than 21 weeks of pregnancy. However, in most cases of primary CMV infection, reliable detection is possible from the 17th week of pregnancy, provided that at least six weeks have passed since maternal infection [26,27]. If infection occurs later in pregnancy, detection of the virus from amniotic fluid should be attempted at least 6 weeks after maternal infection [70]. While PCR and viral isolation are widely recommended in cases of primary maternal infection, [6,26,27,29,31,69], there is no consensus regarding testing in non-primary infections. Although the overall risk of fetal damage is lower in such cases, when damage does occur it can be severe. Maternal treatment with high-dose valaciclovir has been shown to reduce the risk of fetal infection by approximately 60–70% [26,27]. Antenatal diagnosis should therefore be considered even in cases of non-primary infection, particularly when infection occurrs during the first trimester and/or when fetal abnormalities are detected [26].

## 8. Can Fetal Infection and Damage Severity Be Predicted?

As stated earlier, the simplest and most accurate method for assessing fetal infection is molecular detection of viral DNA in the amniotic fluid [26,27,69]. However, this is an invasive procedure. Tanimura and Yamada [71] reported that CMV DNA can also be detected in uterine cervical secretions, a non-invasive procedure; however, its diagnostic accuracy has not yet been established.

Ultrasonographic findings in the second or third trimesters may help identify possible fetal damage and estimate its extent, but they are not diagnostic because they are observed in fewer than half of infected fetuses [53,54,60,65,72,73]. The viral load in the amniotic fluid often reflects the severity of fetal infection and may be used for predicting the possible degree of fetal damage. Quantitative determination of CMV DNA in the amniotic fluid showing ≥ 1000 genome equivalents provided 100% sensitivity for detecting fetal infection. Viral loads of ≥100,000 genome equivalents (copies/mL of amniotic fluid) predicted the development of symptomatic infection [74]. Viral load in maternal blood is also a relatively good predictor. A similar relationship was reported for amniotic fluid viral load by Gilad et al. [38] in a meta-analysis of eight studies. A higher viral load was associated with a higher risk of cCMV. Ozdemir et al. found that 82% of fetuses with PCR-positive CMV DNA in the amniotic fluid had central nervous system abnormalities [75].

There were other attempts to predict fetal damage with partial success. Vorontsov et al. found that retinoic acid receptor responder 2 (RARRES2, chemerin), an immunomodulatory protein detected in amniotic fluid, and galectin-3-binding protein (immunomodulatory and important in cell adhesion) are significantly higher in the affected fetuses [76].

However, it seems that the maternal viral load and fetal brain imaging are still the most effective predictors of fetal damage.

It is still unknown why some women transmit the virus to the fetus, while others do not. Mortality is relatively high among neonates with cCMV following primary maternal infection [10], but rare in infants with cCMV born after maternal non-primary infection, even when neurological damage is similar—as well as in asymptomatic infants with primary infection [33]. There is currently no data on mortality among children with cCMV following treatment or among children born to mothers treated with valacyclovir.

## 9. What Are the Ways for Prevention and/or Reduction of Maternal–Fetal CMV Transmission?

### 9.1. Prevention of Exposure

The most effective way is prevention of maternal infection in pregnancy.

Prevention of exposure can be achieved by practicing strict personal hygiene, particularly thorough handwashing with soap and water after contact with diapers or oral secretions, especially when caring for children in daycare [26,60]. Women who develop a mononucleosis-like illness during pregnancy should be evaluated for CMV infection and, if positive, counseled about the possible risks in pregnancy. If a woman has primary CMV infection, particularly with persistent IgM antibodies and/or virus shedding in the urine, it is generally recommended to postpone conception for 3–6 months [26,29]. The optimal interval before subsequent pregnancies remains debated, as Fowler et al. [77] reported rare cases of fetal CMV infection occurring even years after maternal primary infection.

### 9.2. Prevention by Cytomegalovirus Hyperimmune Globulin (CMV HIG): A Less Effective Way with Possible Side Effects

CMV HIG is a preparation enriched with high titters of CMV-specific antibodies. It is typically administered to individuals at high risk for CMV infection, such as organ transplant recipients and immunocompromised patients [78]. CMV HIG is generally administered once a month during pregnancy at a dose of 100 IU/kg.

CMV HIG acts primarily by providing passive immunity through the transfer of high-titer CMV-specific antibodies that neutralize circulating CMV virions, inhibiting viral replication and dissemination. Additionally, HIG may enhance immune-mediated clearance of infected cells through mechanisms such as opsonization and antibody-dependent cellular cytotoxicity (ADCC) [79]. CMV HIG is thought to reduce vertical transmission by limiting viral load in maternal circulation and potentially decreasing viral spread across the placenta. However, clinical studies have shown mixed results regarding its effectiveness [80].

Nigro et al. [79] were the first to suggest that HIG is a potential prenatal therapy for cCMV infection. In their 2005 non-randomized study, Nigro et al. demonstrated that CMV HIG administration to pregnant women with seroconversion reduced the vertical transmission rate from 40% (19/47) in untreated women to 16% (6/37) in those treated. Several later studies indeed supported the potential efficacy of CMV HIG in preventing mother-to-fetus transmission of the virus [81,82,83]. Subsequent studies [84,85,86], failed to demonstrate a significant reduction in transplacental transmission rates following CMV HIG administration in primary infections. However, there were some indications of a reduction in the severity of cCMV symptoms. In addition, complications related to the CMV HIG were noted in a few pregnant women.

Given its uncertain efficacy and potential risks, CMV HIG is not currently recommended as standard therapy. The recent European consensus recommendations [26] advise against routine monthly administration of HIG at a dose of 100 IU/kg. Nevertheless, biweekly administration at a higher dose (200 IU/kg) can be considered in selected cases of very recent primary infections.

### 9.3. Reduction in Viral Fetal Transmission by Maternal Treatment with High Doses of Valacyclovir

Acyclovir and its prodrug valacyclovir are antiviral medications widely used for the treatment of herpes simplex and varicella-zoster infections. Valacyclovir, a prodrug of acyclovir, offers improved bioavailability and more convenient dosing, making it effective in treating herpes zoster and genital herpes [87,88].

Valacyclovir is rapidly converted into its active form—acyclovir—after oral absorption in the digestive tract. Once inside a virally infected cell, acyclovir is phosphorylated by viral thymidine kinase into acyclovir triphosphate, its active metabolite. Because acyclovir has minimal effects on human DNA polymerase, it is considered both effective and well tolerated. Additionally, over 80% of acyclovir is excreted unchanged in the urine, reducing the risk of cellular toxicity [89,90]. In pregnant women, valacyclovir administration results in significantly increased plasma acyclovir levels compared to direct acyclovir administration, with higher peak concentrations and greater overall daily exposure, enhancing its antiviral efficacy [91]. Decades of clinical data have not demonstrated any increase in major birth defects associated with valacyclovir use during pregnancy.

In recent years, an increasing number of studies have demonstrated the effectiveness of valacyclovir in lowering the rate of fetal cytomegalovirus infection during pregnancy [88,92]. In a randomized, double-blind, placebo-controlled trial, Shahar-Nissan et al. [92] evaluated the effectiveness of valacyclovir (8 g/day) in preventing vertical CMV transmission in pregnant women with primary CMV infection during early pregnancy. Only 11% of amniocenteses were CMV-positive in the valacyclovir group compared with 30% in the placebo group, corresponding to a 71% relative reduction in transmission risk. The effect was more pronounced in first-trimester infections, where valacyclovir reduced transmission from 48% in the placebo group to 11%. Treatment was well tolerated, with no significant adverse events reported.

Zammarchi et al. [88] similarly found that in 205 pregnant women treated with valacyclovir, there was a relative reduction in transplacental transmission by 61%, in comparison to 242 untreated women. Furthermore, the rate of symptomatic cCMV infection at birth was reduced by 83%. Maternal side effects were generally mild, with one case of reversible renal toxicity.

A recent meta-analysis [93,94] provided further evidence supporting the effectiveness of valacyclovir in reducing the risk of cCMV when administered during pregnancy. Overall these findings suggest that valacyclovir at high doses of 8 g/day serves as a promising preventive strategy for cCMV, with the strongest benefit observed when initiated early in pregnancy. Valaciclovir administered to a CMV-positive mother was found not only to reduce transplacental passage of the virus but also to decrease viral load in the infected fetal blood [95,96]. These recent clinical trials demonstrating valacyclovir’s effectiveness in reducing CMV transmission should prompt reconsideration of global screening policies [97], as indeed suggested by a group of European experts and others [26,29]. As ongoing studies continue to explore potential antiviral options to reduce the risk of vertical transmission [93], it may now be time to consider expanding the European recommendations to a global scale. It is also interesting to note that a study protocol of the outcome of routine screening for CMV in pregnancy was published by the CITEMB about two years ago [98].

## 10. Is There Effective Treatment of Infants with cCMV?

Currently, two antiviral agents—ganciclovir and its oral prodrug valganciclovir—are considered effective for the treatment of infants with cCMV. Both can also be given orally [30]. Neonatal treatment should be initiated as early as possible [29]—preferably within the first month of life—as initiation beyond this point, up to 14–16 weeks, provides only partial benefit [29]. In one of the first trials, ganciclovir treatment in neonates with symptomatic cCMV prevented hearing deterioration, which was significantly more common in untreated patients [99]. Michaels et al. [100] similarly reported prevention of hearing loss progression in nine treated children with cCMV, although no improvement in existing hearing deficits was observed. Intravenous ganciclovir treatment also had some benefits in neonatal CMV retinitis [101]. In a relatively large trial of 96 neonates with cCMV, treatment with valganciclovir for 6 months was superior to treatment for 6 weeks only. There were no differences in hearing loss, but the 6-month treatment was more efficient in improving long-term neurodevelopmental outcomes [102]. The beneficial effects of 6-month over 6-week treatment was also demonstrated by Dobbins et al. [103]. To date, there is no solid evidence that antiviral treatment improves long-term neurodevelopmental outcomes. Likewise, no proven preventive benefit has been demonstrated in children who are asymptomatic at birth [6,23]. Treatment should last for at least six weeks, but preferably for longer, up to six months. The European expert consensus recommends approximately six weeks of therapy, although treatment durations of six weeks to six months have been reported in clinical practice [26].

## 11. Can Postnatal CMV Transmission via Breast Milk Affect the Newborn Infant?

Unlike cCMV, postnatal CMV (pCMV) infection is generally considered to have limited clinical significance. Most cases are acquired through breastfeeding—a common and typically benign route of transmission. In full-term infants pCMV is usually asymptomatic and is not associated with long-term sequelae, largely due to sufficient transplacental transfer of maternal antibodies and more robust immune responses. Therefore, routine intervention is generally not recommended for full-term newborns. In contrast, pCMV in preterm and low-birth-weight infants can result in substantial clinical complications and may be associated with long-term neurodevelopmental impairments [104]. Thus, pCMV transmission via breast milk represents a meaningful clinical concern, particularly in very-low-birth-weight and preterm populations.

In preterm neonates, pCMV can lead to sepsis-like illness, hematologic abnormalities, hepatitis, pneumonitis and bronchopulmonary dysplasia, particularly in those born before 32 weeks of gestation or weighing less than 1500 g [104]. CMV shedding is detected in up to 90% of breast milk samples from seropositive mothers, yet only about 20% of exposed infants acquire infection.

Several factors modulate transmission risk, including viral load, mucosal integrity and maternal antibody levels. While short-term morbidity is well documented, long-term neurodevelopmental outcomes remain uncertain, with studies showing mixed results. Preventive strategies such as short-term pasteurization of breast milk may effectively reduce viral infectivity while preserving its nutritional benefits. There is no consensus regarding routine antiviral therapy in pCMV [100,101,102].

A systematic review and meta-analysis by Hu et al. [105] assessed the transmission of CMV via breast milk and the effectiveness of various feeding practices in reducing infection among LBW and preterm infants. Analyzing data from 21 studies involving 1920 infants, CMV infection occurred in 19.3% of infants fed untreated breast milk, compared to 13.5% and 9.1% among those receiving frozen or mixed feeding, respectively. Although severe CMV disease was relatively uncommon, the findings underscore the vulnerability of pre-term infants and support the adoption of modified milk-handling practices, using frozen breast milk, minimizing transmission risks. Several investigators studied various aspects of the effects of CMV-positive breast milk on premature infants [106,107,108]. The main conclusions were that the milk viral load correlates with the rate of pCMV. Song et al. [109] recommended routine screening for CMV in the breast milk of mothers who delivered preterm infants. Since the rate of shedding into breast milk in seropositive women is about 80%, it is advised not to feed infants with fresh milk if it is infected by CMV but with frozen milk or a combined diet, as this decreases the rate of CMV transmission to the infant [110].

In summary, postnatal CMV transmission via breast milk is generally benign in full-term infants but may result in significant morbidity in very preterm and very-low-birth-weight neonates. While freezing breast milk reduces viral load and lowers transmission rates, it does not completely eliminate CMV infectivity. Short-term pasteurization appears to be the most effective method for preventing transmission, although it may partially affect some bioactive components of human milk. Therefore, modified milk-handling strategies should be considered in high-risk populations, balancing the proven benefits of breastfeeding with the need to minimize CMV transmission risk.

## 12. Can CMV Infection Be Prevented by Vaccination?

To date, no vaccine for the prevention of CMV disease has been approved for use. So far, most of them have had low effectivity or unaccepted side effects.

### 12.1. Unsuccessful Vaccines

A live attenuated vaccine based on the Towne 125 strain was developed approximately 50 years ago [111], but failed to prevent infection in women of childbearing age who were exposed to CMV-shedding children [112]. Another was developed and appeared safe and immunogenic in early trials [36]. Other unsuccessful approaches included a recombinant CMV vaccine based on the envelope glycoprotein gB, plasmid-based DNA vaccines, [113] and other trials. The unique biology of CMV poses significant challenges to developing a safe and effective vaccine using traditional approaches [114].

### 12.2. Recent Promising Vaccines, Under Clinical Trials

#### 12.2.1. Virus-like Particle Platform

In a recent first-in-human Phase 1 trial, Langley et al. [115] evaluated the safety and immunogenicity of a novel enveloped virus-like particle (eVLP) CMV vaccine in healthy, CMV-seronegative adults. The vaccine was well tolerated, and most adverse events were mild and transient. Participants developed neutralizing antibodies targeting fibroblast and epithelial cell entry pathways and antibodies against the AD-2 epitope of gB, which has been associated with protective immunity. Antibody levels remained elevated six months after the final dose, highlighting the vaccine’s potential to induce durable immunity.

Preliminary reports indicate promising immunogenicity and good tolerability in early-phase trials [116].

Animal model studies have further demonstrated effective protection against cCMV. Collectively, these findings underscore the importance of multivalent VLP vaccine designs incorporating both gB and the pentameric complex to achieve optimal protection against cCMV [109,110].

#### 12.2.2. mRNA Vaccine Platform

One of the most promising candidates in CMV vaccine development is mRNA-1647, a multivalent mRNA-based vaccine developed by Moderna [117,118,119]. This vaccine encodes six CMV antigens—glycoprotein B (gB) and, respectively, the pentameric complex (gH/gL/UL128/UL130/UL131A) —which are essential for viral entry into fibroblasts and epithelial cells, the primary routes of CMV infection and dissemination. In a first-in-human Phase 1 randomized trial, Fierro et al. [118] demonstrated that mRNA-1647 was safe and well tolerated, with no vaccine-related serious adverse events. The vaccine elicited robust, dose-dependent immune responses. These immune responses were sustained for over a year in both CMV-seronegative and seropositive adults [109]. Hu et al. compared mRNA-1647 with the earlier gB/MF59 subunit vaccine. mRNA-1647 induced more durable and functionally superior immune responses, including broader neutralization against multiple cell types and stronger ADCC activity—key mechanisms potentially linked to protection against CMV transmission.

Recent advances, particularly in mRNA and virus-like particle (VLP) platforms, have demonstrated encouraging safety and immunogenicity profiles, reigniting optimism for an effective CMV vaccine. While no candidate has yet received regulatory approval, the progress of mRNA-1647 into Phase 3 trials and the promising results from VLP-based formulations represent major milestones. However, mRNA-1647 has not received regulatory approval, and its safety and efficacy during pregnancy apparently remain unstudied.

In summary: several strategies are currently available to reduce the risk of fetal CMV infection. These include prevention of maternal exposure (avoiding contact with body secretions from infected children), serological screening, viral load ascertainment and maternal valacyclovir treatment to reduce transplacental transmission. Administration of CMV hyperimmune globulin (HIG) is currently not recommended due to limited efficacy and potential side effects. Vaccination represents the most promising long-term preventive measure, but no vaccine has yet been approved. Of all available strategies, maternal valacyclovir treatment appears to be the most effective, reducing transplacental viral transmission by 60–70%.

## 13. The Debate Around Routine CMV Screening in Pregnant Women and in Neonates: What Is the Current Evidence and the Preferred Recommendations?

### 13.1. Screening in Pregnancy

**Figure 2:**
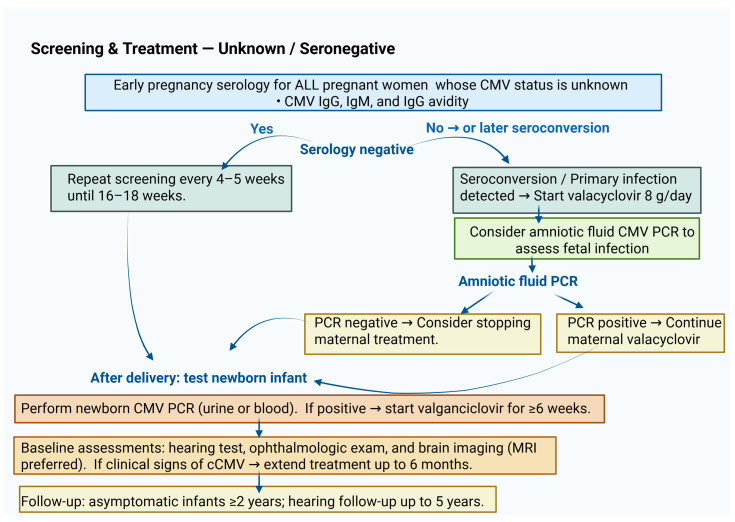
This figure states the recommended policy for screening pregnant women who are CMV seronegative or with unknown serological status and the recommended behavior if seroconversion occurs in the first trimester of pregnancy.

**Figure 3:**
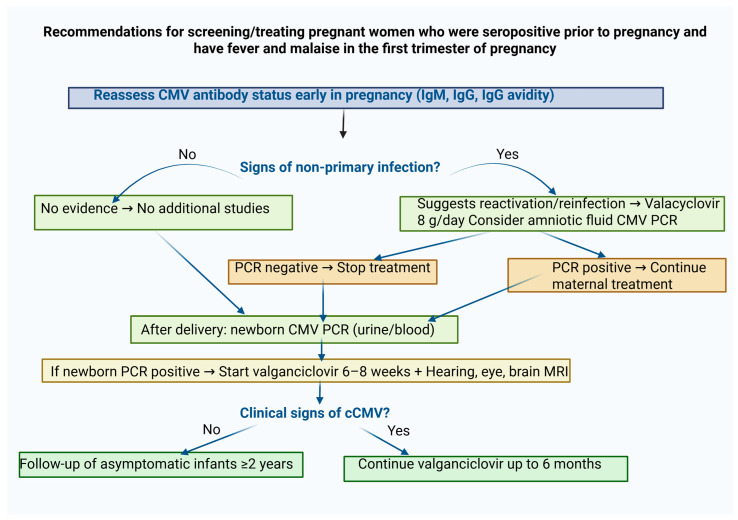
This figure states the recommended policy in pregnant women who are seropositive for CMV prior to pregnancy and have clinical signs of fever and malaise in the first trimester.

Until recently, there was near-universal consensus to refrain from screening pregnant women for CMV. These recommendations relied on the fact that the serological diagnosis was not sufficiently reliable and the lack of effective tools to decrease fetal infection [120]. Economic burden was an additional factor. Thus, even when maternal CMV was proven, the main option was to consider antenatal diagnosis of fetal infection and assess possible damage [6,23,65,66,121]. In recent years, it has become obvious that treatment of infected pregnant women with high daily doses of valacyclovir dramatically reduces the viral transfer to the fetus. This effective therapeutic approach prompted a reconsideration of first-trimester CMV screening. Consequently, the European group of experts, along with others, advocated in 2024 first-trimester serological screening for all women who are CMV-seronegative or have unknown serostatus prior to pregnancy [26]. Their consensus recommendations address almost all facets of CMV infection in pregnancy and emphasize two important recent findings: 1. the highest damage of primary CMV infection is in the first trimester of pregnancy, and 2. antiviral treatment with valacyclovir reduces vertical CMV transmission by about 70%. Based on these findings, the authors recommend that pregnant women should undergo primary CMV screening early in the first trimester, and if seronegative, be re-tested monthly until 14–16 weeks of gestation. They also recommend carrying out IgG avidity testing to be able to differentiate between recent and less recent primary CMV infection. They additionally recommend that all pregnant women with confirmed primary CMV infection receive daily valacyclovir at a dose of 8 g/day (Figure 2 and Figure 3).

Collinet et al. [122] categorized CMV screening strategies into primary, secondary and tertiary prevention frameworks. However, the authors cautioned against associated risks, including increased anxiety, overuse of invasive diagnostics and potential for unnecessary pregnancy terminations. They suggested that universal neonatal screening and integration with hearing screening programs might serve as more practical tertiary prevention strategies, emphasizing the need for accurate epidemiological data and well-defined clinical pathways.

A systematic review by Xie et al. [121] examined 11 international clinical guidelines and two consensus statements, showing that universal maternal CMV screening is still not routinely implemented, largely due to insufficient data and ethical concerns. However, some guidelines support targeted screening for high-risk women, particularly those exposed to young children. The authors call for urgent updates to clinical guidelines considering recent therapeutic advances and the growing body of evidence.

Very recently, Lim et al. even analyzed the cost utility of universal first-trimester screening for CMV and found it to be cost-effective and comparable to the cost utility of other screening methods, such as screening for Down syndrome [65]. Similar conclusions were reported by other investigators [98].

This data shows the advantages of universal CMV screening of women in the first trimester of pregnancy, like the screening for other possible fetal disorders.

#### 13.2. Screening of Newborn Infants

A comprehensive review by Chiopris et al. [23] highlighted key diagnostic challenges: the absence of standardized prenatal screening protocols, limited sensitivity of current diagnostic tools and the high prevalence of asymptomatic infections at birth. While the authors do not support universal screening of pregnant women, they recommend targeted neonatal screening using PCR testing of blood, urine or saliva in infants with suspected cCMV infection, given that routine clinical examinations and newborn hearing screening often fail to identify infected cases. However, the effectiveness of treatment for asymptomatic cases remains unproven and carries risks of hematologic and potential long-term toxicities.

Lantos et al. [123] presented a novel geographically weighted cost-effectiveness analysis evaluating universal newborn screening for cCMV. Using data from over 96,000 infants across seven U.S. metropolitan areas, the authors showed that universal screening reduced severe-to-profound hearing loss and resulted in higher overall cost savings per infant screened. Cost-effective arguments in favor of universal screening hold true even after accounting for geographic variation in prevalence and healthcare access, positioning it as the optimal strategy to reduce CMV-related morbidity and improve long-term child health outcomes.

Most recently, Schleiss and Blázquez-Gamero [124] provided a comprehensive, policy-oriented review of universal newborn CMV screening, proposing it as the emerging standard of care, as it seems to be more effective and cost-efficient than hearing evaluation-targeted approaches. However, they also cautioned against overmedicalization, overtreatment and psychosocial stress on families, particularly in cases of clinically inapparent infection. They also call for dual maternal–newborn screening models.

In summary, recent evidence underscores the importance of early detection strategies for cCMV, with growing support for universal first-trimester maternal screening as well as newborn screening, given their clinical benefits, cost-effectiveness and potential to reduce health disparities. These recommendations remain relevant as long as no safe and effective CMV vaccine is available.

## 14. Conclusions

CMV is considered the most teratogenic human virus, capable of affecting the developing embryo and fetus following primary and non-primary maternal infection. The resulting damage may manifest or progress during the early postnatal years, affecting neurological functions such as hearing, vision and intellectual development. Because of the absence of typical clinical findings, both maternal infection and cCMV in the newborn infant often go undetected, complicating efforts to prevent or mitigate damage. Both maternal valaciclovir and neonatal valganciclovir are effective in reducing viral load, thereby decreasing transplacental transmission or the severity of neurological sequelae in the newborn. Hence, the time has come to implement routine first-trimester screening for recent maternal CMV infection, as well as universal newborn screening to detect all CMV-positive infants, including those with minimal or no symptoms at birth. CMV-positive infants should undergo longitudinal neurodevelopmental follow-up and hearing evaluation during the first several years of life. Such policies should remain in place until an effective CMV vaccine becomes available.

## Figures and Tables

**Table 1:** Fetal CMV infection and auditory system damage.

Study/Author	Main Findings	Implication for Hearing
Teissier et al. (2011) [40]	CMV-infected inner ear structures in all fetuses studied, with infection severity correlating with CNS damage.	Disruption of potassium homeostasis in the inner ear may drive sensory cell degeneration and result in SNHL.
Gabrielli et al. (2013) [42]	CMV infected the inner ear in 45% of fetuses, especially the stria vascularis and organ of Corti.	Inner ear infection can lead to SNHL even in the absence of brain ultrasound abnormalities.
Bartlett et al. (2017) [48]	Asymptomatic infants exhibited 7–11% hearing loss compared with 34–41% in symptomatic infants.	No reliable viral marker predicts outcome; consistent follow-up until school age is recommended for both symptomatic and asymptomatic children.
Hranilovich et al. (2020) [45]	MRI abnormalities were significantly associated with failed newborn hearing screening and early onset hearing loss.	Brain MRI should be considered part of the evaluation of infants with cCMV, even if asymptomatic at birth.
Craeghs et al. (2021) [44]	Brain abnormalities correlate with early hearing loss (84% specificity, 43% sensitivity).	Neuroimaging can identify infants at risk for early hearing loss.
Corazziet al. (2022) [47]	Children with cCMV often show vestibular and postural disorders.	Vestibular impairment can occur independently of hearing loss, underscoring the importance of assessing both systems.
Kabani et al. (2023) [46]	Viral load in urine and saliva is higher in symptomatic infants	Viral load alone is insufficient to predict hearing loss.
Keymeulen et al. (2023) [49]	Hearing loss occurred in 29.2% of asymptomatic children and in 70.8% of the symptomatic children. Only 70.4% of CMV-infected children had normal development.	Neurodevelopmental issues, including hearing problems, can emerge later. All children with cCMV should receive multidisciplinary neurodevelopmental follow-up.
Smyrli et al. (2024) [50]	Among children asymptomatic at birth, 10-15% developed neurodevelopmental disorders, most commonly SNHL.	The risk of hearing loss in asymptomatic infants is relatively low, but long-term surveillance remains advisable.
Gabrielli et al. (2024) [43]	CMV showed tropism for the auditory pathway, infecting the stria vascularis and activating microglia in the auditory cortex, especially in cases with high brain viral load.	Both peripheral (cochlear) and central (cortical) auditory damage may contribute to CMV-related SNHL.

**Table 2:** Fetal CMV infection and neurodevelopmental problems. Studies presented in Table 1 are not discussed here.

Study/Author	Main Findings	Implication for Neurodevelopment
Ivarsson et al. (1990) [57]	Case report of two children with congenital CMV infection who had severe disabilities, including autism.	The study suggests that autism may be among the neurodevelopmental sequelae of severe cCMV infection.
Boppana et al. (1997) [53]	Among 56 symptomatic cCMV-infected newborns, 70% had abnormal cranial CT scans. 90% of these developed at least one neurodevelopmental sequela.	Abnormal newborn cranial CT is a strong predictor of later neurodevelopmental impairment in symptomatic cCMV, whereas clinical signs alone are unreliable for prognosis.
Noyola et al. (2001) [54]	In children with symptomatic cCMV, microcephaly and abnormal cranial CT at birth were strong predictors of later intellectual and motor disability.	Microcephaly and abnormal neonatal brain imaging strongly predict poor neurodevelopmental outcome. Early head circumference and CT findings can guide prognosis and intervention.
Lipitz et al. (2002) [60]	Among 18 live-born infants with confirmed cCMV, 4 had neurological abnormalities; 3 of these had normal prenatal ultrasound.	Normal prenatal ultrasound does not exclude risk of later neurologic impairment. Long-term neurodevelopmental follow-up is warranted even in apparently normal cCMV cases.
Yamashita et al. (2003) [58]	Out of 7 children with symptomatic cCMV, 2 (28.6%) were later diagnosed with autism with global developmental delays with MRI evidence of impaired myelination.	Findings suggest a potential association between cCMV-related brain injury and subsequent ASD.
Bartlett et al. (2017) [48]	Children with asymptomatic cCMV performed similarly to healthy controls on standardized neurodevelopmental assessments.	Despite overall good outcomes, long-term neurodevelopmental follow-up is recommended as no reliable marker predicts later sequelae.
Craeghs et al. (2021) [44]	Brain MRI was useful for predicting early neurological risk, though not definitive for long-term outcomes.	Even with normal early imaging, continued neurodevelopmental surveillance is essential.
Keymeulen et al. (2023) [49]	In a cohort of 753 children with cCMV, 29.6% had some level of neurodevelopmental impairment. Adverse outcomes were seen in both symptomatic (53.5%) and asymptomatic (17.8%) children.	Neurodevelopmental follow-up is essential for all cCMV-infected children, with particular attention to hypotonia, ASD and speech delays—even in the absence of hearing loss.
Smyrli et al. (2024) [50]	A low rate of children asymptomatic at birth still showed neurodevelopmental impairments later in life.	All cCMV-exposed children should receive long-term neurodevelopmental follow-up.
Salome et al. (2025) [30]	Higher maternal blood CMV viral load was associated with more severe neonatal disease and adverse neurodevelopmental outcomes.	Maternal viral load could be used as an early indicator for identifying infants at risk of neurodevelopmental sequelae, and may serve as an early biomarker for risk stratification.

## Data Availability

No new data was created or analyzed in this study. Data sharing is not applicable to this article.

## Abbreviations

The following abbreviations are used in this manuscript:

CMV	Cytomegalovirus
cCMV	Congenital cytomegalic virus
pCMV	Postnatal Cytomegalovirus
IUGR	Intrauterine Growth Restriction
HIG	Hyperimmune Globulin
ASD	Autism Spectrum Disorder
VLBW	Very Low Birth Weight
eVLP	Enveloped Virus-Like Particle
ADCC	Antibody-Dependent Cellular Cytotoxicity
